# Safety and Efficacy of Anti-CD19-Chimeric Antigen Receptor T Cell Combined With Programmed Cell Death 1 Inhibitor Therapy in a Patient With Refractory Post-Transplant Lymphoproliferative Disease: Case Report and Literature Review

**DOI:** 10.3389/fonc.2021.726134

**Published:** 2021-09-16

**Authors:** Gang Feng, Qing Li, Haibo Zhu, Yanyu Jiang, Jijun Yuan, Yingxin Fu, Qi Deng

**Affiliations:** ^1^Department of Kidney Transplantation, Tianjin First Central Hospital, School of Medicine, Nankai University, Tianjin, China; ^2^Department of Hematology, Tianjin First Central Hospital, School of Medicine, Nankai University, Tianjin, China; ^3^Shanghai Genbase Biotechnology Co. Ltd., Shanghai, China

**Keywords:** post-transplant lymphoproliferative disease, diffuse large B-cell lymphoma, programmed cell death 1, chimeric antigen receptor, T cells

## Abstract

Post-transplant lymphoproliferative disease (PTLD) often exhibits poor prognosis and high mortality, and there are no uniform guidelines for the treatment of this disease. Anti-CD19 chimeric antigen receptor (CAR) T cells show significant efficacy in treatment of relapse/refractory diffuse large B-cell lymphoma (DLBCL). Treatment using anti-CD19-CAR T-cell therapy in PTLD has been limited by immunosuppressants and has not been widely employed. In this study, a refractory post kidney transplant DLBCL patient with a high tumor burden was enrolled in a clinical trial of anti-CD19-CAR T-cell therapy. The tacrolimus dose was not decreased during combination chemotherapy, as the creatinine level of the patient increased. To improve the function of autologous T cells, combination therapy with anti-CD19-CAR T cells and programmed cell death 1 (PD-1) inhibitors was selected. After treatment with the combination therapy, the patient was diagnosed with grade 1 cytokine release syndrome and grade 3 immune effector cell-associated neurotoxicity syndrome. The amplification peak of anti-CD19-CAR T cells reached 9.01% on day 7. With PD-1 inhibitor maintenance therapy, his disease was maintained in partial remission for 18 weeks. However, his tumor suddenly increased in size, and he discontinued the treatment, including radiation therapy. The anti-CD19-CAR T cell and PD-1 inhibitors have a combined effect on PTLD, and this combination therapy needs to be further explored.

## Introduction

Post-transplant lymphoproliferative disease (PTLD) is a common complication after solid organ transplantation (SOT) (1). PTLD is a group of heterogeneous lesions characterized by uncontrolled proliferation of lymphocytes due to immunosuppression (2) and includes a variety of histopathological types, ranging from reactive polyclonal B-cell benign proliferation to malignant invasive lymphoma. Malignant and invasive lymphoma types have rapid progression, poor prognosis, and high mortality (3). More than 70% of PTLD cases are associated with Epstein-Barr virus (EBV) infection (4). Owing to the heterogeneity of PTLD and the lack of prospective studies, there are no uniform guidelines for the treatment of this disease. Reduction of immunosuppressive therapy is the first and most important step in the treatment of PTLD and should be started as early as possible (5). Rituximab, an anti-CD20 monoclonal antibody, has become the standard treatment for patients with PTLD who do not respond to immunosuppressant reduction (6–8). Chemotherapy is usually required as a concurrent or sequential therapy in typical PTLD, whereas surgical resection or radiotherapy might be used as adjuvant treatment (9, 10). However, some patients with PTLD develop relapsed/refractory (R/R) disease after standard combination therapy. In particular, PTLD patients with high tumor burden diagnosed with diffuse large B-cell lymphoma (DLBCL) have a poor prognosis (1).

Anti-CD19 chimeric antigen receptor (CAR) T-cell therapy has shown remarkable effects in R/R non-hodgkin lymphoma, especially in R/R DLBCL (11, 12). However, some R/R DLBCL patients, particularly those with a high tumor burden, responded poorly to this therapy (13). Treatment of PTLD using anti-CD19-CAR T-cell therapy has been limited by immunosuppressants and has not been widely employed. Programmed cell death 1 (PD-1) is an important central checkpoint in tumor progression (14). A recent study showed that overexpression of the *PD-1* gene in DLBCL is associated with high tumor aggressiveness (15). Moreover, immunomodulatory agents such as PD-1 inhibitors may be used in patients with transplant-related malignancies. However, the problems of transplantation immunosuppression and cancer immunoregulation caused by PD-1 inhibitor therapy must be addressed.

Here, we report the successful use of anti-CD19-CAR T cell with PD-1 inhibitor therapy followed by PD-1 inhibitor maintenance therapy conducted after kidney transplantation in a patient diagnosed with DLBCL who had a refractory case of PTLD with high tumor burden. After the combined treatment, the patient achieved partial remission despite undergoing continued treatment with immunosuppressives.

## Case Description

A 46-year-old patient (deceased) received allogeneic kidney transplantation owing to a 10-year history of chronic kidney disease (stage 5) in July 2006. Baliximab was used at the induction of immunosuppression therapy for kidney transplantation. For immunosuppressive therapy after kidney transplantation, we administered 150 mg bid oral cyclosporine, 750 mg bid myfortic, and 20 mg Qd prednisone. The serum creatinine level decreased to 120 μmol/L after the transplantation. However, on the sixth day after the operation, the patient developed acute rejection with a serum creatinine level of 556 μmol/L. The immunosuppressive therapy was changed from 20 mg Qd prednisone to 250 mg Qd intravenous methylprednisolone for three consecutive days and from 150 mg bid cyclosporin to 150 mg bid tacrolimus. The patient’s serum creatinine level declined to 129 μmol/L 1 month after the operation, and he successfully recovered. After discharge, the immunosuppressive therapy included the following doses: 2.5 mg bid tacrolimus, 750 mg bid myfortic, and 15 mg Qd prednisone. Subsequently, the patient underwent regular outpatient reexamination and showed stable kidney transplant function.

**Figure 1:**
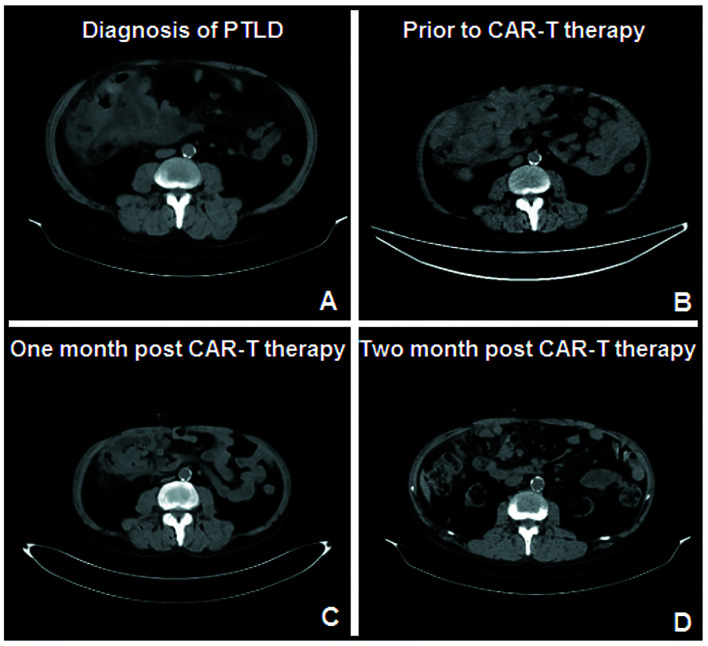
Results of the abdominal CT throughout the course of treatment. **(A)** Large area of mixed density in the right middle and lower abdomen when the patient was diagnosed with PTLD. **(B)** Before the anti-CD19-CAR T cell combined with PD-1 inhibitor therapy, he was refractory to all the combined chemotherapies. **(C, D)**. One and 2 months after the combination therapy, CT scan showed a significantly smaller abdominal mass.

In July 2020, the patient was admitted to our hospital with bloating and abdominal pain for 15 days. A large, irregularly shaped, untender mass was observed on his abdomen. Computed tomography (CT) of the abdomen revealed a large area of mixed density in the right middle and lower abdomen (**Figure 1A**). Laparoscopic biopsies of the mesentery and greater omentum were performed. Pathological results indicated germinal center B-cell-DLBCL, EBV-, and a Ki67 index >85%. The positive rate of MYC detected by fluorescence *in situ* hybridization was 70%, whereas the rates for Bcl-2, Bcl-6, and TP53 were negative. No abnormal B-lymphocyte phenotype was found in the bone marrow by flow cytometry (FCM); however, 4.78% abnormal B lymphocytes were found in drainage from the right lung pleural fluid. He was diagnosed with PTLD, germinal center B-cell-DLBCL (a histological classification of PTLD), stage IV, EBV-, with an international prognostic index score of 4.

Mycophenolate mofetil was immediately discontinued in July 2020. Tacrolimus and prednisone doses were reduced from 4 mg/day and 20 mg/day to 1 mg/day and 10 mg/day, respectively. He received two cycles of frontline therapy with rituximab, cyclophosphamide, vincristine, doxorubicin, and prednisone (R-CHOP). However, his symptoms of abdominal distension and pain were not relieved, and CT still showed an enlarged abdominal mass. Subsequently, he underwent two cycles of R-CHOP combined with etoposide (R-ECHOP). However, the abdominal mass only showed a slight reduction in size after four cycles of chemotherapy. Rituximab combined with bendamustine, etoposide, vincristine, and methylprednisolone (R-BEVD) and rituximab combined with etoposide, carboplatin, and ifosfamide (R-ICE) were chosen as salvage therapies. However, the patient was refractory to all these combined chemotherapies (**Figure 1B**) and was therefore diagnosed with refractory PTLD. The tacrolimus dose was maintained at 1 mg/day during combination chemotherapy because of the increased creatinine levels.

**Figure 2:**
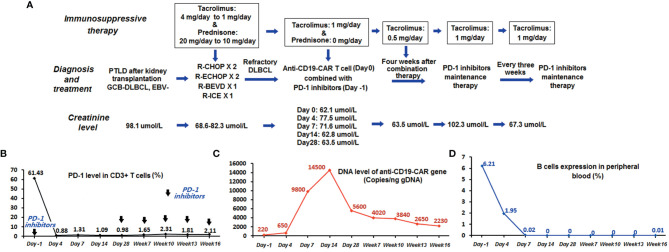
Monitoring the course of anti-CD19-CAR T cell combined with PD-1 inhibitor therapy. **(A)** Flowchart of combination and maintenance therapy. **(B)** The expression level of PD-1 in CD3+ T cells in peripheral blood detected by FCM declined to less than 2%. **(C)** The expression level of the anti-CD19-CAR gene remained more than 10 times higher than the baseline level before therapy. **(D)** The patient had a persistent deficiency of B lymphocytes in his peripheral blood.

The patient accepted our recommendation for treatment of refractory PTLD. He was enrolled in a clinical trial of anti-CD19-CAR T-cell therapy (*ChiCTR1800019622*) as a refractory DLBCL patient and signed an informed consent form. The expression of PD-1 in CD3+ T cells in the peripheral blood was 61.43% at the time of enrollment. Peripheral blood mononuclear cells (PBMCs) for the anti-CD19-CAR T-cell therapy were collected by leukapheresis and isolated by Ficoll density gradient centrifugation. Immunosuppressive therapy for the kidney transplant was adjusted again on the day of leukapheresis. Prednisone was discontinued, and the tacrolimus dose was maintained at 1 mg/day. He received lymphodepleting chemotherapy with fludarabine (30 mg/m^2^) and cyclophosphamide (400 mg/m^2^) from day -4 to day -2. Thereafter, he received PD-1 inhibitors (Sintilimab, 200 mg) on day -1. On the 12th day of cultivation, transduction efficiencies of anti-CD19-CAR were analyzed by FCM. The efficiency of anti-CD19-CAR transduction in this patient was 44.17%. The proportion of anti-CD19-CAR T cells on the harvest date was 5.38 × 10^6^ cells/kg. Autologous humanized anti-CD19-CAR T cells were infused on day 0 (2 × 10^6^ cells/kg) in December 2020 (**Figure 2A**).

Adverse events (AEs) manifested as fever up to 37.8°C with chills on day 1 after anti-CD19-CAR T-cell infusion, accompanied by fatigue, dizziness, headache, and weakness. The highest temperature recorded was 38.4°C on day 7. He was diagnosed with grade 1 cytokine release syndrome (16) and grade 3 immune effector cell-associated neurotoxicity syndrome (17), which was relieved on day 14 with symptomatic treatments only. Anti-CD19-CAR T-cell expression level in CD3+ T cells in peripheral blood was detected by FCM on days 0, 4, 7, 14, and 28. The anti-CD19-CAR T-cell amplification peak reached 9.01% on day 7 (**Table 1**). There were no abnormal peaks of cytokines (including interleukin-6, -2R, or -10 or tumor necrosis factor-α) in the peripheral blood, as detected using enzyme-linked immunosorbent assay. Meanwhile, the proportion of CD3+ CD8+ T cells detected by FCM reached a peak of 79.65% on day 28, and CD19+ B-cell aplasia continued up to week 12 (**Table 1**). The DNA expression level of the anti-CD19-CAR gene detected by quantitative PCR reached its peak at 14500 copies/ng gDNA on day 14. The expression level of PD-1 in CD3+ T cells in peripheral blood detected by FCM declined to less than 2% during the combination therapy (**Figure 2B**).

**Table 1:** Side effects during anti-CD19-CAR T cell combined with PD-1 inhibitor therapy.

	Prior therapy	Day 4	Day 7	Day 14	Day 28
**CAR-T(%)**	0.00	0.67	9.01	3.94	0.83
**IL-6(U/ml)**	3.10	7.02	13.76	6.05	3.13
**IL-2R(pg/ml)**	1.27	0.47	5.48	2.3	1.05
**IL-10(pg/ml)**	0.25	0.19	8.1	2.79	1.21
**TNF-α(pg/ml)**	1.02	0.89	4.07	1.35	1.36
**IFN-γ(pg/ml)**	0.56	0.89	5.15	2.30	1.78
**CD3+T(%)**	95.8	95.4	93.7	89.4	90.31
**CD3+CD4+T(%)**	44.72	30.21	16.63	15.53	13.71
**CD3+CD8+T(%)**	52.42	62.25	72.38	70.47	79.65
**Treg(%)**	6.02	6.12	8.41	10.98	7.05
**Ferritin(ng/ml)**	1285	1720	1180	979	621
**Hb(g/L)**	107	112	108	106	107
**WBC(×10^9^/L)**	4.05	5.2	7.23	8.35	6.52
**PLT(×10^9^/L)**	103	95	34	229	240

Kidney function of the patient remained stable, while immunosuppressive therapy for the kidney transplant was maintained with tacrolimus at a dose of 1 mg/day. His creatinine level rose to 102.3 μmol/L when the tacrolimus dose was reduced to 0.5 mg/day 4 weeks after the anti-CD19-CAR T cell + PD-1 inhibitor therapy. He was diagnosed with acute rejection and was readministered a 1 mg/day dose of tacrolimus. Subsequently, his renal function gradually declined (**Figure 2A**).

Between 1 and 2 months after the combination therapy, all his symptoms were relieved. The CT scan showed a significantly smaller abdominal mass than before (**Figures 1C, D**). The patient achieved partial remission 1 month after combination therapy, according to the Lugano Revised Criteria for Response Assessment (18).

Because the dose for immunosuppressive therapy could not be reduced owing to the kidney transplant, the patient received maintenance therapy with PD-1 inhibitors (Sintilimab, 200 mg) every 3 weeks starting at 28 days after the anti-CD19-CAR T-cell infusion to avoid further progression of the disease. The expression level of the anti-CD19-CAR gene detected 16 weeks after cell infusion remained more than 10 times higher than the baseline level before therapy (**Figure 2C**). The expression level of PD-1 in the peripheral blood remained below 3% throughout this period. The patient exhibited persistent B-lymphocyte deficiency in his peripheral blood (**Figure 2D**). During maintenance therapy (PD-1 inhibitors and tacrolimus 1 mg/day for 3 weeks), we observed a continued reduction in tumor volume (**Figure 1**). His disease was in partial remission for 18 weeks. However, his tumor then suddenly increased in size, and the patient did not elect to receive further treatment, including radiation therapy. When he was diagnosed with progressive disease and refused further treatment, his creatinine level was 98.6 μmol/L. The proportion of anti-CD19-CAR T cells within the CD3+ T-cell population in the peripheral blood was 0% at this time. Because of the rapid progression of the disease, we did not have the opportunity to obtain additional data on CD19 expression in the tumor tissue.

## Discussion

The incidence of PTLD has increased as the number of SOT patients have increased. However, survival time has been simultaneously prolonged. In addition, an increasing number of cases of very late PTLD onset can occur more than 20 years after SOT (19). EBV infection, immunosuppression status, and genetic susceptibility are important factors for PTLD (20, 21). Increased malignancy and rapid progression are characteristics of EBV-negative PTLD, which usually develops several years after SOT (22, 23). In the EBV-negative PTLD patient in this study, the disease was refractory to conventional chemotherapy and progressed rapidly.

Although anti-CD19-CAR T-cell therapy has achieved impressive results in R/R DLBCL, there are still many patients who do not benefit from this therapy. Patients with high tumor burden or immunodeficiency have poor response to chemotherapeutic drugs and immunotherapy (13, 24). Our patient exhibited EBV-negative PTLD, a high tumor burden, refractoriness to chemotherapeutic therapy, including rituximab, and was given continuous immunosuppressive therapy, all of which were disadvantages for the prospects of anti-CD19-CAR T-cell therapy.

The tumor microenvironment plays a major role in preventing durable responses to immunotherapy in hematologic malignancies (25). Macrophages, myeloid-derived suppressor cells, and regulatory T cells in the tumor microenvironment are major inhibitors of immunosuppression in anti-CD19 CAR T-cell therapy (26, 27). Patients with the highest PD-1/PD-L1 interaction scores did not respond to anti-CD19-CAR T-cell therapy or relapsed within 3–6 months (12). Anti-CD19 CAR T cells derived from T cells with high PD-1 expression result in failure of this therapy (28). Fortunately, PD-1 inhibitors combined with anti-CD19 CAR T cells may overcome such immunosuppressive effects (29). Our study also demonstrated the synergistic effects of PD-1 inhibitors combined with chemotherapeutic regimens or anti-CD19-CAR T cells in R/R B-cell lymphoma (30, 31). Some biological theoretical studies have been reported (32), providing a basis for the application of PD-1 inhibitors in DLBCL patients with MYC overexpression. Therefore, for this MYC-overexpressing R/R DLBCL patient, we predicted that PD-1 inhibitors might serve as an effective maintenance therapy after anti-CD19 CAR T-cell therapy. When the patient was enrolled in our clinical trial, the expression of PD-1 in the peripheral blood was 61.4%, and the positive rate of MYC was 70%. These are important factors associated with the poor efficacy of anti-CD19-CAR T-cell therapy. Therefore, we selected a combination therapy of anti-CD19-CAR T cells and PD-1 inhibitors. Although there was some benefit from the combination therapy, the proportion of anti-CD19-CAR T cells within the CD3+ T cell pool in the peripheral blood declined to 0.83% 28 days after CAR T-cell infusion. The rapid decline in the proportion of anti-CD19-CAR T cells might be one of the reasons for disease progression in this patient.

The infusion of PD-1 inhibitors following transplantation led to complete loss of the allograft in an animal model study (33). Another animal study suggested that PD-1 blockade aggravated the progression of EBV+ PTLD (34). Therefore, we encourage the use of additional agents for transplant recipients with tumors in clinical settings (35). However, some studies on the use of PD-1 inhibitors in organ transplant patients without rejection have been reported (36–38). A study was conducted on 69 cancer patients with kidney transplants receiving immune checkpoint inhibitors (39). Following this therapy, 42% of patients developed acute rejection, while 28% lost their allograft. No significant renal damage occurred throughout the course of treatment, although rejection occurred due to tacrolimus dose reduction. In this case, there was no significant rejection of PD-1 inhibitor therapy, which might be related to tacrolimus therapy. PD-1 inhibitors are associated with a high risk of rejection in patients with PTLD but may also lead to improved PTLD outcomes.

AEs were another problem that affected the prognosis for this patient. AEs associated with PD-1 inhibitors are immune-associated events in the respiratory and circulatory systems (28). Fortunately, the safety of the combination therapy and subsequent PD-1 inhibitor maintenance therapy were acceptable. There were no interruptions in any of the therapies due to AEs. In particular, kidney function of the patient remained stable throughout the combination therapy. Until the disease progressed after the combination therapy, his creatinine level did not increase significantly.

His immunosuppressive therapy was maintained with tacrolimus 1 mg/day during the combination therapy and maintenance therapy. However, immunosuppressive therapy may lead to the recurrence of PTLD. Considering the patient history, we decided to continue immunosuppressive therapy with tacrolimus; meanwhile, we chose the PD-1 inhibitors in combination therapy and the subsequent maintenance therapy. We anticipated that the PD-1 inhibitors would overcome the immunosuppressive effects and enhance T-cell function (28) while simultaneously increasing the risk for acute rejection. Fortunately, there was no serious acute rejection due to increased T-cell function throughout the course of therapy. In contrast, the humoral immune response mediated by B cells plays an important role in rejection after SOT (40, 41). We hypothesized that the deficiency of B cells induced by anti-CD19-CAR T-cell therapy might prohibit acute rejection and protect renal function.

Previous studies have reported the efficacy and safety of anti-CD19-CAR T-cell therapy in R/R PTLD patients (42–46) (**Table 2**). All refractory PTLD patients to date received anti-CD19-CAR T-cell therapy with discontinuation or reduction in the dose of immunosuppressive therapy. They either did not respond to anti-CD19-CAR T-cell therapy or had disease progression within a few months after responding to anti-CD19-CAR T-cell therapy. Therefore, maintaining the efficacy of anti-CD19-CAR T-cell therapy is a problem that needs to be solved. None of these refractory PTLD patients received maintenance therapy after anti-CD19-CAR T-cell therapy.

**Table 2:** Summary of PTLD cases received CD19 CAR-T therapy.

Patient	Age	Sex	SOT, years	Time of PTLD diagnosis	EBV tumor status	Pathology	Therapy before CAR-T	Immunosuppressive therapy in CAR-T	CRS	ICANS	Outcome	Maintenance therapy after CAR-T	Ref.
**Patient 1**	54	M	Kidney, Pancreas	20 years	Negative	DLBCL	RIS, Rituximan, R-CHOP, R-ICE	None	Grade 1	Grade 2	SD, death at day 115	None	(39)
**Patient 2**	54	F	Heart	27 years	Negative	DLBCL	RIS, R-CHOP, R-ICE	Lower dose of Sirolimus	Grade 2	Grade 3	PD, death at day 44	None	(39)
**Patient 3**	71	M	Kidney	10 years	Negative	DLBCL	RIS, R-CHOP, R-DHAX, Ibrutinib	Lower dose of Prednisone	Grade 2	Grade 4	PD, Death at day 15	None	(39)
**Patient 4**	38	M	Kidney	10 years	Negative	DLBCL	RIS, R-CHOP, R-GEM-Ox	Prednisone (5 mg/day)	Grade 1	None	CR, Lasts up to +28 weeks	None	(40)
**Patient 5**	44	M	Kidney	10 years	Negative	DLBCL	RIS, R-CHOP	None	Grade 1	Grade 3	CR, Refractory at +34 weeks.	None	(40)
**Patient 6**	41	M	Kidney	7 years	Negative	DLBCL	R-EPOCH, R-GDP, R-ESHAP, Pola+BR	None	None	None	PR, PD at +12 weeks	None	(40)
**Patient 7**	69	M	Kidney	25 years	Negative	DLBCL	R-POCH, R-GDP	Lower dose of Tacrolimus	Grade 1	None	CR, Refractory at 3 months	None	(41)
**Patient 8**	50	F	Kidney	5 years	Negative	DLBCL	R-CHOP, R-ICE, ASCT	Lower dose of Tacrolimus	Grade 2	None	CR, Lasts up to 9 months	None	(41)
**Patient 9**	66	M	Liver	8 years	Negative	DLBCL	R-HOP, ICE	Lower dose of Tacrolimus	Grade 1	None	PR, Lasts up to 3 months	None	(41)
**Patient 10**	4	M	Liver	21 months	Positive	BL-PTLD	R+CTX+ Methylprednis	None	Grade 2	None	CR, Lasts up to 16 months	None	(42)
**Patient 11**	17	F	Heart	4 months	Positive	DLBCL	RIS, R-COP, R-COPADM, R-CYVE, O-ICE	Lower dose of Tacrolimus and Prednisone	Grade 1	none	CR, Lasts up to 6 months.	Plan to undergo HCT	(43)

PTLD, posttransplant lymphoproliferative disorders; DLBCL, diffuse large B cell lymphoma; EBV, Epstein Barr virus; CRS, cytokine release syndrome; ICANS, immune effector cell-associated neurotoxicity syndrome; RIS, reduction in immunosuppression; R, rituximab; CHOP, cyclophosphamide, vincristine, doxorubicin, and prednisone; DHAX, dexamethasone, cytarabine, and oxaliplatin; ICE, ifosfamide, carboplatin, and etoposide; EPOCH, etoposide, adriamycin, vincristine, cyclophosphamide, prednisone; GDP, gemcitabine, cisplatin, dexamethasone; ESHAP, etoposide, methylprednisolone, cisplatin, cytarabine; Pola+BR, polatuzumab vedotin, bendamustine, rituximab; COPADM, cyclophosphamide, vincristine, prednisone, doxorubicin, and high dose methotrexate; O-ICE, obinutuzumab-ICE; CYVE, cytarabine, etoposide; ASCT, autologous stem cell transplantation; HCT, hematopoietic cell transplantation.

In our study, despite many obstacles, the patient achieved partial remission as a result of the anti-CD19-CAR T cell + PD-1 inhibitor therapy and subsequent PD-1 inhibitor maintenance therapy. The selection of PD-1 inhibitors might have antagonized the immunosuppressive effect of T cells and the overexpression of MYC in tumor cells. Combining anti-CD19-CAR T cells and PD-1 inhibitors as well as the application of tacrolimus might reduce the risk of acute rejection caused by PD-1 inhibitors. Although the refractory PTLD in this patient eventually progressed, this combination therapy could be attempted again with more success.

## Data Availability Statement

The raw data supporting the conclusions of this article will be made available by the authors, without undue reservation.

## Ethics Statement

This study was approved by the Medical Ethics Committee of the Department of Hematology, Tianjin First Center Hospital (Tianjin, China) (approved no. of ethic committee: 2018N105KY). The patients/participants provided their written informed consent to participate in this study. Written informed consent was obtained from the individual(s) for the publication of any potentially identifiable images or data included in this article.

## Author Contributions

Concept and design: QD and YF. Drafted or revised the manuscript: GF and QL. Acquisition of data: HZ and YJ. Analysis and interpretation of data: JY. Writing, review, and/or revision of manuscript: GF. Study supervision: QD. All authors contributed to the article and approved the submitted version.

## Funding

This work was supported by a grant (81970654) from National Natural Science Foundation of China to YF.

## Conflict of Interest

JY was employed by the company Shanghai Genbase Biotechnology Co., Ltd.

The remaining authors declare that the research was conducted in the absence of any commercial or financial relationships that could be construed as a potential conflict of interest.

## Publisher’s Note

All claims expressed in this article are solely those of the authors and do not necessarily represent those of their affiliated organizations, or those of the publisher, the editors and the reviewers. Any product that may be evaluated in this article, or claim that may be made by its manufacturer, is not guaranteed or endorsed by the publisher.
